# Nonlinear Predictive Threshold Model for Real-Time Abnormal Gait Detection

**DOI:** 10.1155/2018/4750104

**Published:** 2018-06-26

**Authors:** Masoud Hemmatpour, Renato Ferrero, Filippo Gandino, Bartolomeo Montrucchio, Maurizio Rebaudengo

**Affiliations:** Dipartimento di Automatica e Informatica, Politecnico di Torino, Turin, Italy

## Abstract

Falls are critical events for human health due to the associated risk of physical and psychological injuries. Several fall-related systems have been developed in order to reduce injuries. Among them, fall-risk prediction systems are one of the most promising approaches, as they strive to predict a fall before its occurrence. A category of fall-risk prediction systems evaluates balance and muscle strength through some clinical functional assessment tests, while other prediction systems investigate the recognition of abnormal gait patterns to predict a fall in real time. The main contribution of this paper is a nonlinear model of user gait in combination with a threshold-based classification in order to recognize abnormal gait patterns with low complexity and high accuracy. In addition, a dataset with realistic parameters is prepared to simulate abnormal walks and to evaluate fall prediction methods. The accelerometer and gyroscope sensors available in a smartphone have been exploited to create the dataset. The proposed approach has been implemented and compared with the state-of-the-art approaches showing that it is able to predict an abnormal walk with a higher accuracy (93.5%) and a higher efficiency (up to 3.5 faster) than other feasible approaches.

## 1. Introduction

Gait, that is, the manner of walking, is a unique personal trait that recognizes every individual [1]. By revealing the personal trait, gait analysis has numerous applications such as identification, authentication, and action recognition [2–4]. Abnormal gait detection is used in particular in pathological and rehabilitation processes, clinical assessment, automated surveillance, and falling systems [5–7]. In order to decrease fall occurrence and minimize their effects, international organizations advise improving health-care systems. This study focuses on abnormal gait/walk detection with the goal of predicting a fall risk. Fall-risk prediction out of abnormal gait detection is based on the relation between abnormal gait and risk of fall [8].

Critical consequences of falls include long-term disability, reduced mobility, injury, and even death. Physical injury due to a fall, in particular hip fracture, involves economical effects for the society [9]. Several fall risk factors have been identified but few successful approaches are adopted in real cases. Fall-related systems can be categorized into three different types: fall detection, prediction, and prevention systems.

Fall detection systems notify a user's associate (e.g., caregivers or acquaintance) in case of fall occurrence [10–15]. These systems can be used to provide fast help after a fall, but they do not avoid it, so they are less effective than other systems. Several fall-risk prediction systems (FPSs) have been developed to estimate future fall risk through some clinical assessments. These tests often involve questionnaires or functional assessments of posture, gait, cognition, and other risk factors. These clinical assessments are subjective and qualitative and typically use threshold assessment scores to categorize people as fallers and nonfallers. Another kind of FPS aims to identify an abnormal gait pattern in order to estimate the probability of a real-time fall occurrence [16–19]. Real-time systems continuously assess the fall risk while the user is doing its daily activity. Fall prevention systems predict a fall and provide a solution for preventing real-time or future falls [20, 21]. Fall prevention systems are helpful in reducing the financial and health consequences of a fall [22].

Both prediction and prevention systems usually have some fundamental steps in common, in which prediction and prevention terms are interleaved: first, data are collected from sensors and are analyzed to compute the appropriate feature set; then, the risk of a possible fall is evaluated through some classification algorithms. Movement sensors are usually exploited to investigate the extrinsic parameters of a fall. In this study, common sensors, such as accelerometers (for measuring the acceleration) and gyroscopes (for measuring the angular rate around one or more axes of the space) are considered since they are easily accessible and do not disturb the privacy of the user (e.g., camera and sound sensors). The popularity of smartphones, the preciseness of the features extracted from embedded sensors [23–25], and their ability to easily engage to the IoT framework, motivates researchers to exploit smartphones in their studies. Although some studies employ additional sensors [18, 19], they bring inconvenience to the users such as wearing augmented shoes, belts, and so on. So, the proposed model only adopts the embedded sensors (i.e., accelerometer) in a smartphone to be convenient for the users.

Most of the FPSs analyze the user's posture or gait variables to investigate the characteristics of the movement. They typically exploit heavy mathematics which leads to high computational time. Since the complexity of an algorithm directly affects the performance, the trade-off between accuracy and complexity is an important consideration. In this paper, a Nonlinear AutoRegressive eXogenous (NARX) walking model is proposed in order to achieve both low complexity and high accuracy. This study is focused on the use of raw accelerometer data for real-time fall-risk prediction to analyze user's data and assess fall risk during the walk. In the preliminary step, normal walking is modeled and stored based on the user's acceleration signals out of the NARX identification technique. Then, the system monitors a sliding window of user acceleration and predicts the future behavior of the user gait in real time. If the predicted output can be distinguished from the normal gait pattern through a specified threshold, the system counts the gait as an abnormal walk. Result shows that this approach has higher accuracy up to 2.8% and lower computational time up to 3.5 times in comparison with the state-of-the-art approaches. In this study, the proposed method detects an abnormal gait while the user is walking in a normal manner on flat surface with casual footwear. The focus is finding a method with high accuracy and low performance in order to be feasible in real cases.

The remainder of the paper is organized as follows: Related works in the literature and complexities are reviewed in Section 2. The identification techniques are explained in Section 3. Gait-modeling formulation is presented in Section 4. Finally, some conclusions are written in Section 5.

## 2. Background and Related Works

FPSs usually consider kinematic fall factors such as velocity, acceleration, tilt, stability, and symmetry. They exploit movement sensors to investigate the kinematic parameters of fall, that is, the characteristics of the movement of the body. Future fall-risk prediction systems estimate the user's fall risk in an offline test such as Timed Up and Go (TUG), Berg Balance Scale (BBS), Sit To Stand (STS), and One-Leg Stand (OLS) [26–29]. If the fall risk is high, a probable future fall can be prevented through some exercises [30]. Typically, mentioned fall risk tests are applied by experts in clinical environments to evaluate balance and lower limb strength.

In the TUG test, the user is asked to rise from an arm chair, walk three metres away, turn, walk back to the chair, and sit down again. A performance higher than a threshold is identified as high risk of falls. However, the suggested threshold value varies in the literature [29].

The BBS test measures user's balance by assessing the performance in carrying out a given task. It comprises a set of simple balance tasks. The degree of success in each task is given by a score, and the final measure is the sum of all scores [28].

In the STS test, firstly, the user sits in the middle of a chair, and he/she places his/her hands on the opposite shoulder crossed at the wrists. Then, he/she keeps the feet flat on the floor. Afterwards, he/she rises to a full standing position and then he/she sits back down again. Eventually, this test counts the number of full stands that are completed in 30 seconds. Since slowing of postural movements can be an indicator of the weak lower limb, a low number of full stands shows a high level of fall risk [26].

The OLS test is used to evaluate the user's balance. The user stands, then he/she raises one leg, with the foot approximately six inches off the ground, keeping the foot raised up for 30 seconds. He/she can sway side-to-side or back-and-forth while maintaining the one-leg stand position. If a user puts his/her foot down some times during the 30-second period, then he/she will fail the test [27].

The drawback of the abovementioned tests is that they demand time and effort. These tests have to be conducted in a supervised environment and may therefore suffer from influences such as the Hawthorne effect, that is, the reaction in which the individual modifies his/her behavior due to his/her awareness of being observed. Unlike the clinical assessment tests, a real-time fall risk is predicted by recognizing an abnormal walking pattern, then the user is alerted [17–19, 31], or an external aid such as a walker or robot is exploited to prevent the fall [20, 21].

In the kinematic analysis of the real-time fall-risk prediction, some approaches detect abnormal gait for predicting a fall risk [17, 32, 33], while others consider the near-fall (i.e., stumble) instances [34]. It is conceivable because the near fall is an indication of a fall. However, a time analysis is missing in order to measure the time between the near-fall detection and the real-fall occurrence. This analysis is essential to check the feasibility of performing any recovery mechanisms before the fall occurrence. However, the main features adopted to detect abnormal gait/walk are explained [34]. In the following formulas, *A*(*t*) indicates the acceleration and *A*_*x*_(*t*), *A*_*y*_(*t*), and *A*_*z*_(*t*) refer to its components along the 3 axes.

When a user significantly tilts in a direction, he/she assumes an abnormal posture, which can lead to a fall. The user tilt is evaluated with the combination of a gyroscope and an accelerometer [17].

*Hjorth parameters* and *energy* measurements of the tilt vector are computed as features of the user gait. Hjorth parameters are statistical measures of the signal, based on its variance var(*A*(*t*)) in time domain [35]:

- Hjorth  activity=var(*A*(*t*)) indicates the signal power.
- $\text{Hjorth mobility}=\sqrt{\left(\mathrm{var}\left(A^{\prime }\left(t\right)\right)/\mathrm{var}\left(A\left(t\right)\right)\right)}$ shows the smoothness of the signal curve.
- Hjorth  complexity=(mobility(*A*′(*t*))/mobility(*A*(*t*))) measures the irregularities in the frequency domain.

The *energy* of the acceleration signal specifies the amount of activity in the vertical and horizontal directions. It estimates the strength of the contact with the floor, so it can be used to recognize an abnormal walk pattern such as stumbling. The energy of the signal is computed as follows:

(1) $$\begin{array}{c} E_{x}={\int^{}}_{-\infty }^{\infty }{\left|A\left(t\right)\right|}^{2}\;\;dt. \end{array}$$

Acceleration-derived features can be used as a near-fall indicator [34]. Several features of the acceleration signal are presented in the following.

*Signal magnitude area* (SMA) is used to classify the user activities. It is computed as

(2) $$\begin{array}{c} \text{SMA}=\frac{1}{T}\left({\int^{}}_{0}^{T}\left|A_{x}\left(t\right)\right|\;\;dt+{\int^{}}_{0}^{T}\left|A_{y}\left(t\right)\right|\;\;dt+{\int^{}}_{0}^{T}\left|A_{z}\left(t\right)\right|\;\;dt\right), \end{array}$$

where *T* is the monitored interval.

*Signal magnitude vector* (SMV) specifies the degree of the movement intensity and the resultant of the acceleration signal as follows:

(3) $$\begin{array}{c} \text{SMV}=\frac{1}{n}\sum_{i=1}^{n}\sqrt{A_{x}^{2}\left(t\right)+A_{y}^{2}\left(t\right)+A_{z}^{2}\left(t\right)}, \end{array}$$

where *n* is the number of samples.

The peak and peak-to-peak are useful measurements of data changing over time. The peak is the maximum value of the signal over the period of time, and the peak-to-peak is the difference between minimum and maximum values of the signal. Moreover, the derivative *A*′(*t*) of the acceleration can be used to indicate the vibration of a user movement. Another feature used in FPSs relies on the relative frequency in data distribution [32].

A co-occurrence matrix is a square matrix that shows the scattering of similar adjacent values at a given offset. Normally, it is applied to the image [36], but its application in fall-risk prediction has been investigated to analyze the acceleration and gyroscope values [32]. Let *p*_*ij*_ be the (*i*, *j*)th element of the co-occurrence matrix to the sum of all the elements. *Contrast*, *homogeneity*, *correlation*, *uniformity*, and *maximum*  *probability* of the co-occurrence matrix [36] are used as features of the user gait [32]:

- *Contrast* shows the intensity between a cell and its neighbors:

(4) $$\begin{array}{c} \text{contrast}=\overset{K}{\underset{i=1}{\sum }}\overset{K}{\underset{j=1}{\sum }}{\left(i-j\right)}^{2}p_{ij}. \end{array}$$

(ii) *Homogeneity* measures the spatial closeness of the distribution of elements to the diagonal of the co-occurrence matrix:

(5) $$\begin{array}{c} \text{homogeneity}=\overset{K}{\underset{i=1}{\sum }}\overset{K}{\underset{j=1}{\sum }}\frac{p_{ij}}{1+\left|i-j\right|}. \end{array}$$

(iii) *Correlation* shows how a value is related to its neighbors:

(6) $$\begin{array}{c} \text{correlation}=\overset{K}{\underset{i=1}{\sum }}\overset{K}{\underset{j=1}{\sum }}\frac{\left(i-m_{r}\right)\left(j-m_{c}\right)p_{ij}}{\sigma_{r}\sigma_{c}}, \end{array}$$

where *m*_*r*_, *m*_*c*_, *σ*_*r*_ and *σ*_*s*_ are computed as follows:

(7) $$\begin{array}{c} \begin{array}{l} m_{r}=\sum_{i=1}^{K}\sum_{j=1}^{K}ip_{ij},\;m_{c}=\overset{K}{\underset{j=1}{\sum }}\overset{K}{\underset{i=1}{\sum }}jp_{ij},\\ \sigma_{r}=\overset{K}{\underset{i=1}{\sum }}\overset{K}{\underset{j=1}{\sum }}{\left(i-m_{r}\right)}^{2}p_{ij,}\;\sigma_{c}=\overset{K}{\underset{j=1}{\sum }}\overset{K}{\underset{i=1}{\sum }}{\left(i-m_{c}\right)}^{2}p_{ij}. \end{array} \end{array}$$

(iv) *Uniformity* computes the sum of the squares of the elements:

(8) $$\begin{array}{c} \text{uniformity}=\overset{K}{\underset{i=1}{\sum }}\overset{K}{\underset{j=1}{\sum }}p_{ij}^{2}. \end{array}$$

(v) *Maximum probability* measures the highest value of the co-occurrence matrix.

The principal component analysis is a mathematical procedure based on an orthogonal transformation of data. The output of the transformation from the accelerometer and gyroscope signals is a set of eigenvectors, which can be used as a feature of walking [33].

### 2.1. Complexity Analysis

The complexity of an algorithm can be presented as a numerical function of the input (*n*) to the algorithm. In this section, the time complexity of the fall-risk prediction algorithms (i.e., feature extraction and the classification) are discussed [17, 32–34].

A combination of average, fixed size matrix multiplication, variance, derivative, and trapezoidal rule is used to compute the feature set [17]. The dominant complexity among the mentioned computation is *O*(*n*). Moreover, decision tree (DT) is adopted to predict a fall. The complexity of the DT classification is related to the number of analyzed features: it is *O*(*v*  *n*⁡log(*n*))+*O*(*n*(log*n*)^2^) plus the complexity of the classification algorithm that depends on the depth of the built tree [37]. In the presented analysis, *v* is the number of features in the decision tree and *n* is the input size of the algorithm.

The best feature set of acceleration signal for abnormal gait detection is extracted from derivative, trapezoidal rule, peak-to-peak, and average [34]. Among these computations, peak-to-peak exploration has the highest complexity equal to *O*(*n*log*n*).

The co-occurrence matrix, average, relative frequency, and standard deviation are adopted to compute the feature set and a neural network to detect abnormal walk [32]. The dominant complexity of the feature set extraction is the calculation time for the co-occurrence matrix which is *O*(*n*^2^) [38], and the complexity of the neural network is *O*(*LW*^2^), where *L* is the number of layers and *W* is the average number of nodes in each layer.

Principal component analysis of the co-occurrence matrix is adopted as a feature set, and random forest classification is exploited to classify the normal and abnormal walks [33]. The classification complexity of this study increases by a factor of *m* (the number of decision trees in random forest) respecting [17] due to the adoption of random forest classification instead of decision tree.

## 3. ARX and NARX Identification

An identification technique is commonly used in many engineering areas to build a function that finds a relation between variables in a system. Generally speaking, finding a mathematical model that adequately describes a system is part of the system identification. Most of the physical activities in nature can be described by a dynamic system, where the output depends on the input history. System identification adopts statistical methods to build a mathematical model from measured data of the dynamic system. A three-step procedure is used in preparing the model: firstly collecting data, then building a convenient model, and finally validating the model. In more detail, the mathematical model is based on a function that depends on a set of unknown parameters *θ*:

(9) $$\begin{array}{c} y=f\left(w,\theta \right), \end{array}$$

where *w* represents the input and output history and *θ* specifies the coefficient parameters. A model can describe either a linear or nonlinear system. In general, the estimation of nonlinear systems is more complex than linear models due to the complex relation among the variables of the model.

AutoRegressive eXogenous (ARX) is a simple, frequently used identification model based on linear difference equation [39]. It describes the input effects on the process output as follows:

(10) $$\begin{array}{c} y\left(t\right)+y\left(t-1\right)+\cdots +y\left(t-na\right)=b_{1}u\left(t-nk\right)+\cdots +b_{nb}u\left(t-nk-nb+1\right), \end{array}$$

where the current output *y*(*t*) relates to a finite number of past outputs *y*(*t* − *k*) and inputs *u*(*t* − *k*). The model is thus entirely defined by three integers *na*, *nb*, and *nk*, where *na* is the number of poles and *nb*−1 is the number of zeros, while *nk* is the pure time-delay in the system.

NARX is a system identification method for discrete nonlinear systems exploiting past input and output data [40]. The NARX model regresses the next output with the previous values of input and output. The mathematical expression of a NARX model is

(11) $$\begin{array}{c} \begin{array}{l} y\left(t+1\right)=f^{o}\left(w^{t}\right),\\ w^{t}=\left[y\left(t\right),y\left(t-1\right),\ldots ,y\left(t-n\right);u\left(t\right),u\left(t-1\right),\ldots ,u\left(t-m\right)\right], \end{array} \end{array}$$

where *u*(*t*) and *y*(*t*) represent input and output values of the system at time step *t*; *m* and *n* represent the input and output delays, and *f*^*o*^ represents a nonlinear mapping function.

(12) $$\begin{array}{c} f^{o}\in \psi \left(\theta \right)=\left\{f\left(w,\theta \right)=\overset{r}{\underset{i=1}{\sum }}\alpha_{i}\sigma \left(w,\beta_{i}\right)\right\}, \end{array}$$

where *θ* is the set of unknown coefficients ([*α*_1_ … *α*_*r*_, *β*_1_ … *β*_*r*_]^*T*^), and *σ* is a *basis function*. Every continuous function can be represented as a linear combination of a particular function called *basis function*. The main problem is finding a good estimation of function *f*^*o*^ with low identification error. Since each estimation has some noise *ε*, a general equation can be presented as follows:

(13) $$\begin{array}{c} Y=F\left(\theta \right)+D_{\varepsilon }, \end{array}$$

where *Y* is the measured output, *F*(*θ*) is a function of *θ*, and *D*_*ε*_ is the prediction error. Parameter *θ* can be estimated by means of the prediction error (PE) method:

(14) $$\begin{array}{c} {\hat{\theta }}^{\text{LS}}=\arg\;\underset{\theta }{\min}V_{N}\left(\theta \right), \end{array}$$

where *V*_*N*_(*θ*)=(1/*N*)([*Y* − *F*(*θ*)^*T*^[*Y* − *F*(*θ*)]) and LS presents the least square. LS strives to minimize the squares of the errors between real and estimated values. It should be considered that *V*_*N*_(*θ*) is nonconvex so the solution may be a local optimum instead of the absolute one.

**Figure 1:**
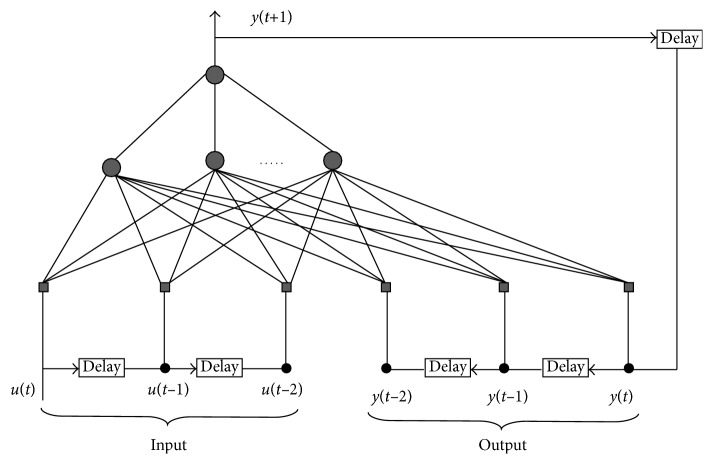
NARX model implementation with neural network.

NARX is based on a neural network to approximate the function *f*^*o*^, as can be seen in Figure 1. The complexity of this part is the same as creating a neural network, but it occurs only once in the training step, and using in real time has a linear complexity.

After preparing an appropriate model, its evaluation is usually done by computing an error over a number of elements, that is, root mean square error (RMSE) and FIT:

(15) $$\begin{array}{c} \begin{array}{l} \text{RMSE}=\sqrt{\frac{1}{n}\overset{n}{\underset{i=1}{\sum }}{\left({\hat{f}}_{i}-f_{i}^{o}\right)}^{2}},\\ \text{FIT}=1-\frac{\text{RMSE}}{\sqrt{\left(1/n\right)\overset{n}{\underset{i=1}{\sum }}{\left({\hat{f}}_{i}-mean\left({\hat{f}}_{i}\right)\right)}^{2}}}, \end{array} \end{array}$$

where *f*^*o*^ is the real system output and $\hat{f}$ is the model output. RMSE measures the error between the measured data and the model. FIT expresses how much the model is fit to the real data. The lowest RMSE and the highest FIT show the best model.

## 4. Gait Modeling Formulation

In this paper, the proposed fall-risk prediction is formulated in three steps: collecting the input/output data, preparing and training the model of normal walk, and calculating the threshold. In the first step, the collected input/output data must be sufficient and representative, so a design of an effective normal/abnormal walk simulation is mandatory. This requires to find an appropriate definition for the abnormal gait. Gait is a cyclic pattern with several consecutive phases which can be formulated using different models [6, 41, 42]. However, defining a normal and subsequently an abnormal gait can be challenging due to the modification of the gait characteristics with aging, type of footwear, and terrain [43–45]. Moreover, gait definition can be altered according to the gait motion observation method, which can consider foot pressure, images, and acceleration. Generally speaking, abnormal gait is different from the usual one, which depends on the personal physical conditions. Abnormal gait can result from genetic anomalies, chronic disease, multiple sclerosis (MS), Parkinson disease, and stroke. In addition, it can be associated with medical conditions such as diminished strength, limited range of motion, poor posture, decreased sensory perception, deformity, wide-based stance, gait variability, stooped postures, freezing of gait, and shuffling gaits [45]. However, an abnormal gait may happen for people with healthy kinematics while doing their daily living activities. A sudden change from normal to abnormal gait can increase the risk of a fall. Detecting such abnormal gaits can help to predict a possible fall. It should be noted that different types of gait behaviors such as running, jumping, and going up/down stairs can be confused with the abnormal gait. Furthermore, typically falls are defined as unintentionally coming down to the floor or lower level [46], and some activities, like stand-to-sitting, sitting on a chair, or lying on a bed, can be confused with real falls. Gait classification methods aim to classify the activities and distinguish such confusing motions [47, 48]. Usually, fall-related studies narrow the monitoring activities under fixed circumstances (e.g., user's age, footwear, and terrain) and strive to detect the abnormal gait patterns which bring high risk of fall for the users [17–19, 34, 49].

A fall may be due to many factors such as weakness, balance deficit, gait deficit, visual deficit, and mobility limitation. Based on these fall factors, the most frequently used methods to simulate abnormal walks are as follows:

- Walking with a straightened knee [17–19]
- Walking with a leg length discrepancy [17–19]
- Walking on a rough surface [49]
- Walking through obstacles [34]

**Figure 2:**
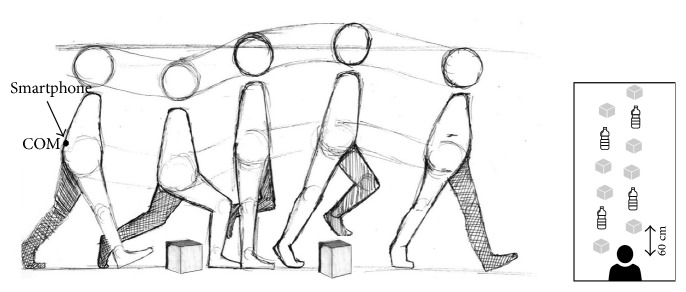
Users walk through obstacles while a smartphone is located in the lower back trunk of the user.

In this paper, an abnormal walk is modeled as irregular gaits obtained by walking through obstacles which can cause stepping, tripping, and stumbling. A flat area with different types of obstacles is prepared. Thirty-one users walked through obstacles without looking at them, as shown in Figure 2. Obstacles included empty boxes (height: 37 cm, length: 20 cm, width: 17 cm) and plastic bottles (height: 20 cm and diameter: 6 cm) which were placed 60 cm far from each other. The users in the experiments are with a weight in the range of 50–110 kg and a height in the range of 157–185 cm. Moreover, they are in the range of 18–68 years old without gait disturbances.

Commonly adopted sampling frequencies range from some dozens to hundreds of Hertz such that they are constant and higher than the gait cycle frequency. In this study, the frequency is fixed to 10Hz. Data are collected through MATLAB R2015b. An iPhone 4S smartphone is adopted in the experiments, equipped with STMicro STM33DH 3-axis accelerometer and STIMicro AGDI 3-axis gyroscope. Since the body center of pressure (COP) reveals several information of user gait, the smartphone is placed on the lower back of the trunk, near the real center of mass (COM) position, assuming that this position moves parallel to the COP, and the same accelerations and positions will be measured [25]. Moreover, three-component acceleration vector describes human movement more precisely [50]. Although, in literature, devices are placed on different positions, the provided accuracy is not always the same [51]. Moreover, it might require a dynamic and automatic calibration of the orientation of the device in case of pocket position [52, 53]. In the current system, users can wear the device without paying attention to its orientation since the device is placed in a predefined position.

Each user walks 10 seconds normally and 10 seconds abnormally (i.e., walking through the obstacles) and he/she repeats the experiments 5 times. In the second step, the NARX model is prepared according to [12]. The proposed model has no input and only the acceleration signal is formulated as system output (*y*(*t*)), so every *u*(*t*) is eliminated. The obtained model is

(16) $$\begin{array}{c} y\left(t\right)=f^{o}\left(w^{t-1}\right),w^{t-1}=\left[y\left(t-1\right),y\left(t-2\right),\ldots ,y\left(t-n\right)\right], \end{array}$$

where *f*^*o*^(*w*)=∑_*i*=1_^*r*^*α*_*i*_*σ*(*w*). The sigmoid function is used as a nonlinear basis function (*σ*). Three samples of normal walk of each user are used to prepare the model. As tuning the parameters of a NARX model is not a straightforward task, further empirical investigation on basis functions and the number of previous data in sliding window (called order) is performed out of a comparative analysis of classification accuracy. Experiments show that 20 basis functions with order equal to 10 achieve higher classification accuracy. Typically, there is a trade-off in higher number of basis functions and order with the performance of the model, so parameters are tuned precisely in order to achieve a good accuracy with low overhead. If the higher number of order and basis function are adopted, the model requires more computational time to predict the future.

**Figure 3:**
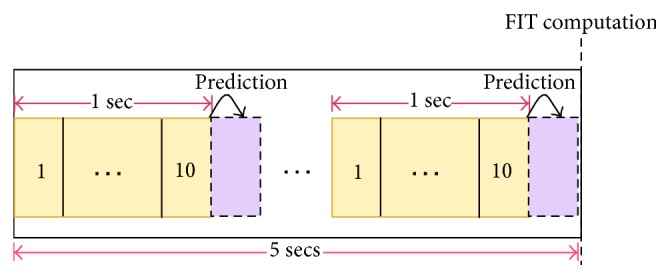
FIT and prediction window slides.

In the third step, to find the appropriate parameter to distinguish normal and abnormal walks, both the FIT and RMSE are computed and compared with the prepared model. The result shows that the FIT is more representative to distinguish normal and abnormal walks. Empirical investigation on the threshold shows that the best accuracy is obtained with a threshold set to 0.02. Eventually, the nonlinear threshold model is ready to be used in the real world. When the user walks, the acceleration values in a sliding window with 10 samples (i.e., 1 second) are obtained to predict the next acceleration value. Then, the FIT is computed with a larger sliding window of 50 samples (i.e., 5 seconds): if the computed FIT is higher than the specified threshold (i.e., 0.02), it is counted as an abnormal walk otherwise as a normal one, as reported in Algorithm 1.  Figure 3 shows an example of the prediction and FIT sliding windows.

**Figure 4:**
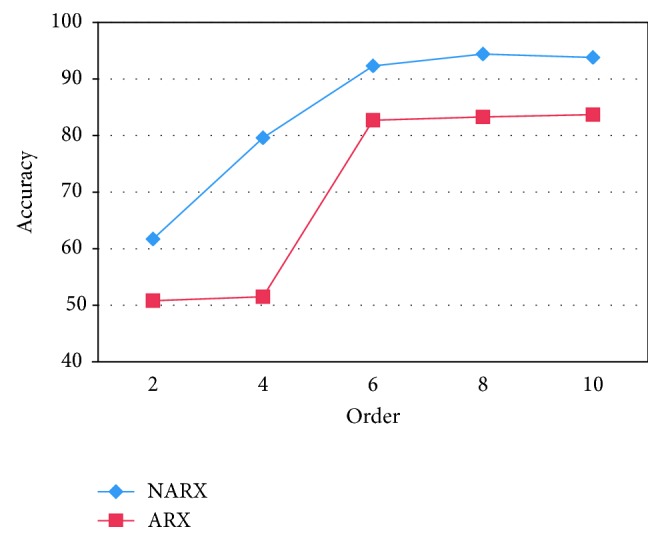
Comparison between ARX and NARX models.

Since the linear identification model has lower overhead than the presented nonlinear one, the same procedure was performed based on the linear ARX model. The result shows that the accuracy of linear regression is lower than the nonlinear one, as can be seen in Figure 4. Therefore, it motivates the use of nonlinear walking model.

In order to evaluate the proposed model with the existing FPSs, described in Section 2, a comparison of accuracy is presented in Table 1. *Accuracy* of an algorithm computes the number of samples correctly classified. *Error rate* is the number of wrong classifications. *Sensitivity* measures the rate of abnormal instances that are correctly identified as abnormal. *Specificity* measures the rate of normal instances that are correctly identified as normal. *Generality* is computed as 1−*Specificity*. 10-fold cross validation is adopted in the presented results. As the result shows, the proposed model has the highest accuracy in abnormal walking pattern recognition.

**Figure 5:**
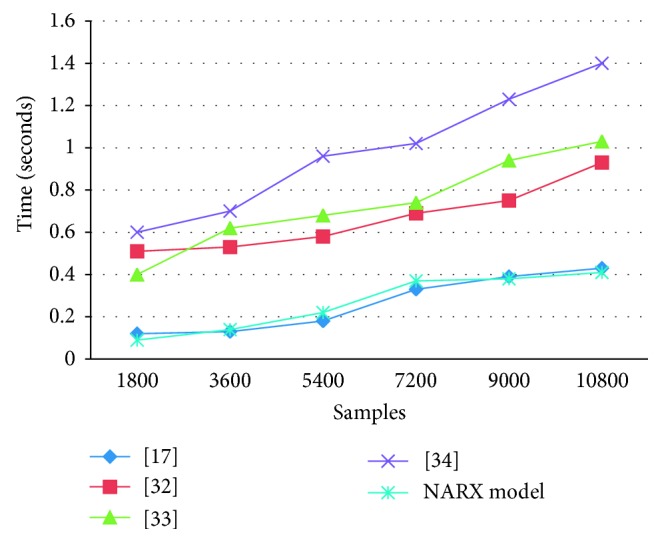
Computational time of different real-time fall-risk predictions.

Further experiments are performed to investigate the computational time of different approaches. In the experimental setup, 10800 samples of normal and abnormal walks are given to different methods. The computational overhead including the feature extraction and classification algorithm of each system is measured cumulatively. The result at different samples is presented in Figure 5. The computational overhead of [32, 33] is significantly high due to the co-occurrence matrix features extraction. Profiling on [34] and [17] reveals that the overhead is mainly due to the local peak computation during feature extraction. Majumder et al. [17] have approximately the same computational time as the proposed method due to its simple feature set; however, it has lower accuracy. The proposed model does not extract heavy feature set; consequently, it has low computational time. Computational time also influences the battery life: for example, executing a machine learning algorithm can run at most 3 hours on a commercial smartphone with a fully charged battery [17]. As such, choosing the right algorithm with a minimal number of features would dramatically decrease the usage of processor and would save energy [54].

An important point in a fall-risk prediction system is its detection time, that is, the time between the starting of the abnormal gait and its detection. A short detection time is important to have sufficient time to perform a supportive mechanism for the user. Further analysis on the proposed method shows that this approach can detect abnormal gait in less than a second after its occurrence. The low complexity of the proposed model with sufficient accuracy fits it for devices with limited resources.

## 5. Conclusion and Future Work

This paper has analyzed various features based on data obtained from the accelerometer and gyroscope extrapolated in fall-risk prediction systems. A NARX model on normal walk is created, and a threshold-based algorithm is proposed to classify normal and abnormal walks. The main considerations in the proposed model are complexity and accuracy. Furthermore, the fall-risk prediction algorithms have been experimentally evaluated based on accuracy and algorithm complexity. The algorithms have been evaluated with a dataset based on 31 users. Based on the presented results, the proposed model has the lowest computational time with the highest accuracy among the presented fall-risk prediction algorithms, and it is able to detect an abnormal gait in less than one second.

The aim of the present line of work is to eventually make a contribution in a generic fall-risk prediction system. All findings of this paper are considered as the building blocks for the future design. More challenging abnormal-like activities consisting in running, jumping, and going up/down will be modeled. In the future work, we plan to recognize the hazardous level of abnormal gait by means of duration and intensity of the abnormality. This would allow proper prevention strategies to avoid serious injuries in case of hazardous situation.

## Figures and Tables

**Algorithm 1:**
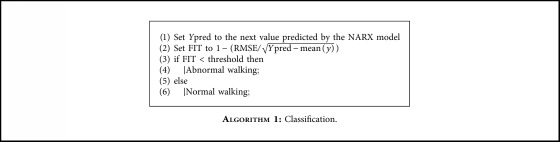
: Classification.

**Table 1:** Result of comparison.

Measures	[17]	[32]	[33]	[34]	NARX model
Accuracy	76.5	82.0	86.2	90.7	93.55
Error rate	23.4	17.9	13.7	9.2	6.45
Sensitivity	81.4	72.4	87.6	93.2	90.9
Generality	28.3	8.3	15.2	11.6	0.03
